# Additive Manufacturing of Biobased Material Used in Electrical Insulation: Comparative Studies on Various Printing Technologies

**DOI:** 10.3390/polym17162248

**Published:** 2025-08-20

**Authors:** Robert Sekula, Alexander Leis, Anne Wassong, Annsophie Preuss, Hermann Hanning, Jan Kemnitzer, Marco Wimmer, Maciej Kuniewski, Pawel Mikrut

**Affiliations:** 1 Hitachi Energy Research, Pawia 7, 31-154 Krakow, Poland; 2 Dressler Group GmbH, 53340 Meckenheim, Germany; leis@dressler-group.com (A.L.); wassong@dressler-group.com (A.W.); preuss@dressler-group.com (A.P.); hermann.hanning@flux-engineering.com (H.H.); 3 Fraunhofer Institute for Manufacturing Engineering and Automation IPA, Universitätsstraße 9, 95447 Bayreuth, Germany; jan.felix.kemnitzer@ipa.fraunhofer.de (J.K.); marco.wimmer@ipa.fraunhofer.de (M.W.); 4 Department of Electrical and Power Engineering, AGH University, 30-059 Kraków, Poland; maciek@agh.edu.pl (M.K.); pawel.mikrut@agh.edu.pl (P.M.)

**Keywords:** power transformers, cellulose electrical insulation, biomaterials, 3DE printing, additive manufacturing, laser sintering, high speed sintering

## Abstract

In the power industry, various electrically insulating materials are used to ensure proper mechanical, thermal, and dielectric performance over decades of equipment operation. In power transformers, cellulose is the predominant material in manufacturing various insulation components. Most of these products are manufactured by wet-molding technology. However, this process is long, labor-intensive, and highly energy-demanding. Under the frame of an EU-funded grant, a new kind of insulation material and manufacturing process were developed. Fully bio-based material (produced in the form of pellets) can be processed using additive manufacturing, allowing for much shorter manufacturing times for insulation products, with considerably less scrap and energy consumption (due to the elimination of the drying stage). The focus of the project was extrusion additive manufacturing technology, but at a later stage, a biomaterial powder was developed, making it possible to print with other technologies. In the paper, comparative studies on various additive manufacturing techniques of newly developed biopolymers have been presented, including extrusion, High Speed Sintering (HSS), and Selective Laser Sintering (SLS). The applicability of such material in power transformers required extensive testing of various properties. These results are discussed in the paper and include: oil compatibility, volume resistivity measurements, permittivity and dissipation factor measurements, determination of partial discharge inception voltage, partial discharges measurement, and breakdown voltage measurements. Although mechanical properties remain below industrial targets, the pioneering results provide a promising route for unique directions toward more sustainable manufacturing of high-voltage cellulose insulation and ideas for improving the material properties during the printing process.

## 1. Introduction

3D printing or additive manufacturing has revolutionized material science by enabling the creation of complex geometries with minimal waste. Among the most promising developments in this field is the use of biopolymers, particularly cellulose and wood-derived polymers, as sustainable alternatives to petroleum-based plastics. These natural materials offer biodegradability, renewability, and biocompatibility, making them ideal for applications in biomedicine, packaging, and construction. One especially unique application of biomaterials is high-voltage electrical insulation, where cellulose predominates. Cellulose, the most abundant organic polymer on Earth, is a primary structural component of plant cell walls; it is non-toxic, biodegradable, and renewable, making it a prime candidate for sustainable additive manufacturing. However, its poor solubility and thermal stability have historically limited its direct use in additive manufacturing. This is why more research on the development of suitable additives for biomaterials must be conducted to ensure better behavior of such materials under elevated temperatures.

The use of cellulose and wood-derived polymers in additive manufacturing aligns with global efforts to reduce plastic pollution and carbon emissions. A 2024 life-cycle analysis confirmed that wood-based biopolymers such as lignin and cellulose nanofibrils have a significantly lower environmental footprint compared to synthetic polymers [1].

The convergence of biopolymer science and additive manufacturing is opening new frontiers in sustainable manufacturing. Cellulose and wood polymers, once limited by processing challenges, are now at the forefront of eco-innovation thanks to advances in nanotechnology and material engineering. As research continues to refine these materials and their applications, we can expect a future where biodegradable, high-performance 3D-printed products become commonplace across industries.

The paper focuses on the potential application of a newly developed biopolymer in high-voltage (HV) electrical insulation components used in power transformers. At the initial stage, under an EU-funded NOVUM grant (European Commission Horizon 2020/SPIRE, proposal number: 768604, proposal acronym: NOVUM), the biopolymer was developed in a granulated form and evaluated by either Fused Deposition Modelling (FDM) or extrusion. At a later stage, the granulated material was converted to powder, and two other additive manufacturing technologies were tested, namely High-Speed Sintering (HSS) and Selective Laser Sintering (SLS). Both of these additive manufacturing technologies are powder-based. In the SLS process, a thin layer of the powdered material is spread across the building platform, and a laser selectively scans and sinters (fuses) the powder. The platform lowers slightly (according to the required resolution of the printed object), and a new layer of powder is spread. HSS process is similar to SLS, but instead of using a laser to fuse the powder, it uses infrared light and an inkjet print head that spreads infrared-sensitive ink over the powder layer. These pioneering studies provide detailed comparisons of the dielectric performance of the printed samples. Additional tests have been performed for the evaluation of mechanical properties, but detailed results of such investigations are not presented in the paper and will be discussed in future publications. It is noteworthy that extrusion-based additive manufacturing enabled the attainment of tensile strengths up to 35 MPa. In contrast, the High-Speed Sintering (HSS) process yielded a maximum of 6 MPa, while samples fabricated via Selective Laser Sintering (SLS) exhibited tensile strengths of only 4 MPa. These values are substantially lower than those reported for the widely recognized polyamide 12 (PA12). For PA12, tensile strengths exceeding 45 MPa have been documented for both extrusion and SLS techniques; also, values above 45 MPa have been observed for the HSS process. Of course, they may differ by 20 to 25% depending on the type of filler (fibers) and its content in the polymer composite.

## 2. Development of New Material and Its Manufacturing

The material used as high voltage insulation in power transformers should be oil-compatible and characterized by a high electrical withstand strength (above 20 kV/mm). Hitherto, in power transformers, cellulose has been the dominant insulation material, with components produced by time-consuming, labor-intensive, and highly energy-demanding wet-molding technology. In addition, for each product, a dedicated mold must be manufactured, and the molding process generates substantial waste of cellulosic material. All these obstacles related to current technology drove the search for new materials and manufacturing processes based on additive manufacturing. However, most commercial bio-based materials for additive manufacturing show insufficient mechanical performance, especially at elevated temperatures, and their dielectric performance is unsuitable for high-voltage applications. Under the EU-NOVUM project, dozens of cellulose-based material formulations were developed in various forms, such as pastes, filaments, and pellets—and extensively tested to obtain good printability parameters.

Cellulose is a polymeric material derived from various natural raw materials, but it cannot be printed or molded without chemical modification. For better processing, cellulose needs to be modified by esterification or etherification; these are the most common commercial methods in use [2,3]. Cellulose-based, thermally moldable materials in ester form, such as cellulose acetate (CA), cellulose acetate butyrate (CAB), and cellulose acetate propionate (CAP), are commercially available for several industrial applications.

The NOVUM compound was as previously described in another article by the authors [4]. The materials used for manufacturing the cellulose-based compound were cellulose acetate propionate (CAP) (CELLIDOR CP300-13, Albis Plastics GmbH, Hamburg, Germany) with a phthalate-free plasticizer content of 13% and a melt flow rate of 7.5 cm^3^/10 min (210 °C, 2.16 kg) as the polymer matrix, 20 wt.-% microcrystalline cellulose (VIVAPUR 105, JRS Pharma GmbH, Weissenborn, Germany) with an average particle size based on laser diffraction of 15 µm as the cellulosic filler, and reactive epoxidized linseed oil (Lankroflex™ L, Valtris Specialty Chemicals, Independence, OH, USA) as the coupling agent.

The microcellulose (MC) fibers were treated with 5% Lankroflex™ L coupling agent (relative to MC dry weight) using a blade blender. The MC-blend was dried overnight in a heat convection oven at 50 °C. CAP was dried at 80 °C for 2 h before compounding. The MC-blend was compounded with CAP using a co-rotating twin-screw extruder (Berstorff ZE 25 × 33 D, Berstorff GmbH, Hanover, Germany). The extruder zone temperatures ranged from 80 to 205 °C, the speed was 100 rpm, and the material output was 2 kg/h. The extrudate granules were then selected as the final material form. More information regarding the development of that NOVUM material can be found in [5].

## 3. Additive Manufacturing: Extrusion-Based

**Figure 1:**
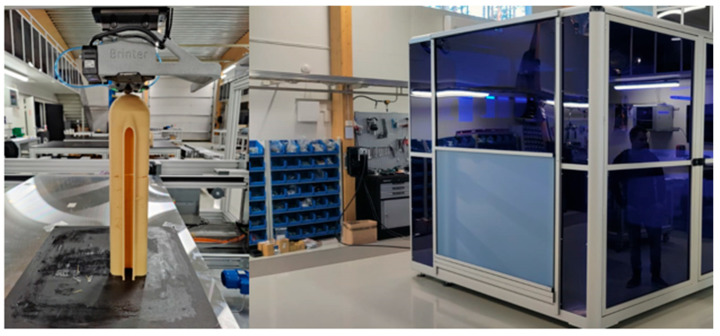
Large-volume-components extrusion-based additive manufacturing machine.

Within the framework of this EU project [5], in addition to bio-material development, a new machine was designed and manufactured by the Britner company in Finland (Figure 1). The printer employs extrusion technology.

Several printing trials were performed using various printing parameters, printing speeds (50, 10, 200, and 300 mm/s), and extrusion nozzles with various diameters (1, 3, and 5 mm). The best printing quality was obtained from the following set of printing parameters:

- Nozzle diameter, 1 mm;
- Layer height, 0.35 mm;
- Tool temperature, 179/206/219 °C (top/middle/nozzle);
- Printing speed, 50 mm/s;
- Printing bed temperature of 40–45 °C with ABS coating.

**Figure 2:**
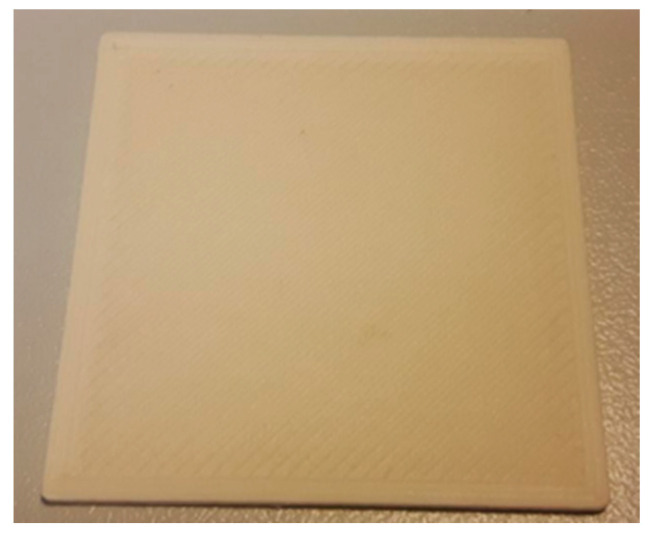
Sample printed via extrusion-based additive manufacturing.

Figure 2 shows a printed flat sample, and a benchmark insulation component was produced by the same FDM method.

**Figure 3:**
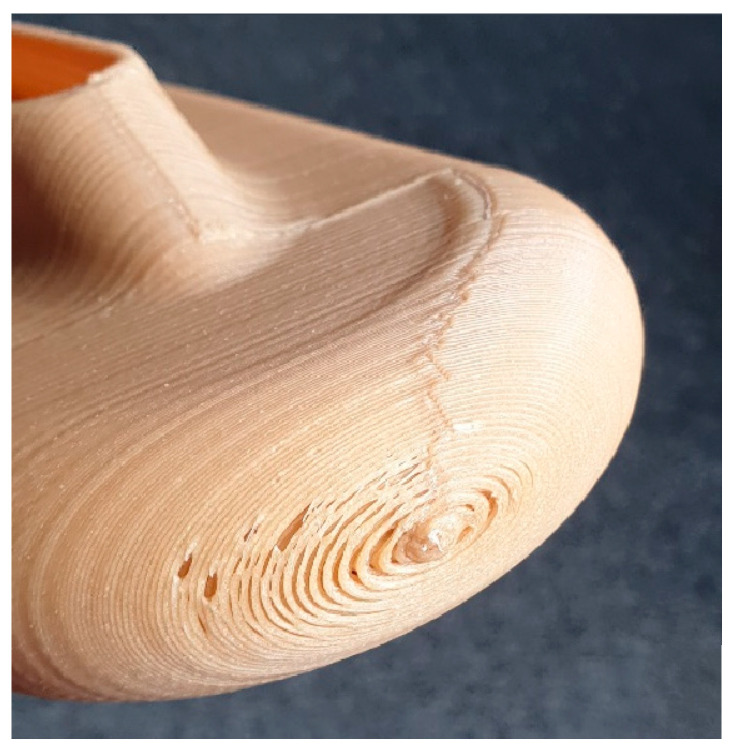
Visible voids after extrusion-based additive manufacturing.

The printing quality was unsatisfactory, mainly due to the presence of external and internal voids (Figure 3), which are unacceptable for electrical insulation purposes. Therefore, it was decided to evaluate other printing technologies such as High-Speed Sintering (HSS) and Selective Laser Sintering (SLS), believing that these printing technologies offer a more uniform microstructure.

As the available material came as granules, there was a need to select appropriate parameters for grinding the granules. This work was carried out by the R@D team at Dressler Group GmbH.

## 4. Powder Production by Grinding

Dressler Group GmbH specializes in the grinding of polymers and refining of polymer powders. With different milling equipment, as well as a selection of flow aids, the aim is to process customer material to an optimized powder for the corresponding end application.

Dressler performed two grinding trials in their technical center, with NOVUM material granules. First, a grinding trial was conducted to evaluate the grindability and to analyze the powder properties. With the results from the first trial, a follow-up trial with a larger raw material quantity was conducted to assess the long-term process stability and to improve the powder properties. Moreover, the content <20 µm of 5 kg powder should be removed to evaluate the influence of fine content on the powder properties.

To micronize tough polymers, Dressler has the possibility to grind materials cryogenically [6]. If amorphous and partly amorphous polymers are cooled down below their glass transition temperature, they become brittle and can be milled more easily. The pilot plants in the technical center make use of this effect.

The particle size distribution was determined via laser diffraction measurements and air classifier sieve measurements at 32 µm, 63 µm, 100 µm, and 160 µm (ISO 4610) [7].

The moisture content of the powder was measured with a halogen moisture analyzer (Kern DBS 60-3, KERN & Sohn GmbH, Balingen-Frommern, Germany).

To analyze the powder properties, the following methods were used: apparent density measurement (DIN EN ISO 60) [8], Hausner ratio measurement (ASTM D1895) [9], funnel flow measurement (DIN EN ISO 6186) [10], and MDK measurement (material development kit, HP, Palo Alto, CA, USA).

Microscope images of the final powder were taken with an optical microscope (Bresser laboratory microscope ETD-201, Bresser, Rhede, Germany).

### 4.1. Laser Diffraction Measurement

The particle size measured by the Horiba Laser is calculated from the detected laser diffraction. This method results, depending on material type and particle shape, in significantly deviating values compared to a sieve. The calculated maximum particle size is much higher in most cases. The results are also dependent on the applied method.

The results are reproducible and therefore suitable for quality control. The benefit of the Horiba laser diffraction is that a full particle size distribution is gained by just one measurement.

### 4.2. Air Classifier Sieve

The air classifier sieve provides a direct physical measurement of the particle size from the weight difference before and after sieving. For example, 100% <150 µm means that 100% of the particles passed through a sieve of 150 µm (Analogous to ISO 4610).

### 4.3. Halogen Moisture Analyzer

The Halogen moisture analyzer measures the moisture content of a sample gravimetrically by drying the sample through heat radiation and determining the loss of mass. Moisture content has a strong influence on powder flowability [11].

### 4.4. Material Development Kit (MDK)

The MDK can be used to simulate the spreadability of a single layer inside an SLS machine, as well as show the influence of temperature on the powder flowability. Coupons with different depths are available, which define the layer thickness of the powder spread. A heating pad below the coupon allows the powder to be heated up to 150 °C. A metal mold with a cutout is used to define the sample amount and form. A metal roller, with a set speed, spreads the powder over the coupon. Then the powder layer is analyzed by an optical unit with a camera, which measures the powder coverage by analyzing the contrast of the powder in comparison to the coupon. For the evaluation, a predefined threshold exists for each coupon. Due to the influence of the powder color on the coverage values (a low contrast results in low coverage values, e.g., in the case of black powders), the spreadability results can only provide a qualitative statement about the powder flow.

### 4.5. Apparent Density Measurement and Hausner Ratio Measurement

The apparent density measurement measures the density of a powder inside a container (in this case, a 100 mL cylinder) without external compaction (1). In contrast to the apparent density, the tapped density measures the density of a powder inside a vessel after being tapped 500 times (2). The Hausner ratio can be used to evaluate the flowability of a powder. It is calculated by dividing the tapped density by the Bulk density. A Hauner ratio <1.2 indicates good flowability.

(1) $$Apparent\;density=\frac{Mass\;of\;material}{Volume\;of\;material\;}$$

(2) $$Tapped\;density=\frac{Mass\;of\;material}{Volume\;of\;material\;}$$

(3) $$Hausner\;ratio=\frac{Tapped\;density}{Bulk\;density}$$

### 4.6. Funnel Flow Test

The funnel flow test measures the time a known amount of powder needs to flow through a funnel of a specific diameter. It is also possible to assess how the powder flows through the funnel.

### 4.7. Grinding Results

After grinding tests number of parameters have been ob-tained and detailed information is provided below.

#### 4.7.1. Particle Size Distribution

For the first grinding trial, the aim was to evaluate the grindability of NOVUM material and to reach a D50 value of 50 to 60 µm.

The targeted specification could be reached, but the final powder contains a high content of fines (19% <32 µm) (Table 1). Many fine particles can negatively influence the flow of a powder. To try and reduce this effect, a coarser milling setting was tested. While it was possible to increase the D50 and D90 values, the D10 value and <32 µm content remained the same. To test the effect of an even finer particle size distribution, a third milling setting (Setting 3) was conducted. Again, the D10 value remained similar, the <32 µm content was increased, as well as the D50 and D90 values.

During the second grinding trial, the targeted specification was predefined by Fraunhofer IPA (Table 2). Due to the brittle nature of NOVUM, it was not possible to reach the desired D10 value during grinding. Therefore, a dedusting step was conducted to raise the D10 value to specification. During the dedusting step, a finer sieve size is used to eliminate fines of a certain size from the final powder. This results in a narrower particle size distribution, but also in material loss.

#### 4.7.2. Powder Properties

To evaluate the powder flowability, the following measurements were conducted: apparent density, Hausner ratio, and funnel test. During the first grinding trial, Setting 1 and Setting 2 (coarse) showed the best flow behavior (Table 3). The Hausner ratio is similar for both powders. Slightly worse behaved was the powder of Setting 3 (fine). No powder was able to flow through a 15 mm funnel.

The powder of the second grinding trial showed a similar Hausner ratio to Setting 1 of the first grinding trial (Table 4). However, the best flow was achieved with the dedusted powder. It has the lowest Hausner ratio and was even able to flow through a 15 mm funnel, after tapping the funnel once.

While the dedusted powder shows the best flow behavior of all the powders, the flow is still poor. To improve the flow even more, a flow aid could be used.

#### 4.7.3. Spreadability

To simulate the spreadability inside an SLS machine and to analyze the effect of temperature on the spreadability of NOVUM-23, MDK measurements were performed. The powder of the first grinding trial was tested with a layer thickness of 500 µm and at 25 °C and 80 °C (Table 5). All three powders showed poor spreading behavior. At both temperatures, but with worse spreadability at the elevated temperature (80 °C). The highest coverage at 25 °C was achieved with the coarse powder (Setting 2).

For the second grinding trial, the spreadability measurements were completed with a layer thickness of 100 µm and 80 °C (Table 6). In comparison to the first grinding trial, both powders showed an increase in spreadability. The reason could be the lower moisture content of the final powders. The spreadability of the dedusted powder was slightly better, even though the coverage value for the non-dedusted powder is higher. This is due to the fact that the powder of the non-dedusted powder piled up at the end of the coupon bed. This powder formation increases the coverage value but is an indication of worse spreading behavior.

To improve the spreadability further, Dressler Group recommended a flow aid trial with dedusted powder. Flow aid can reduce agglomerations inside the powder and therefore improve the powder flow.

#### 4.7.4. Microscope Images

**Figure 4:**
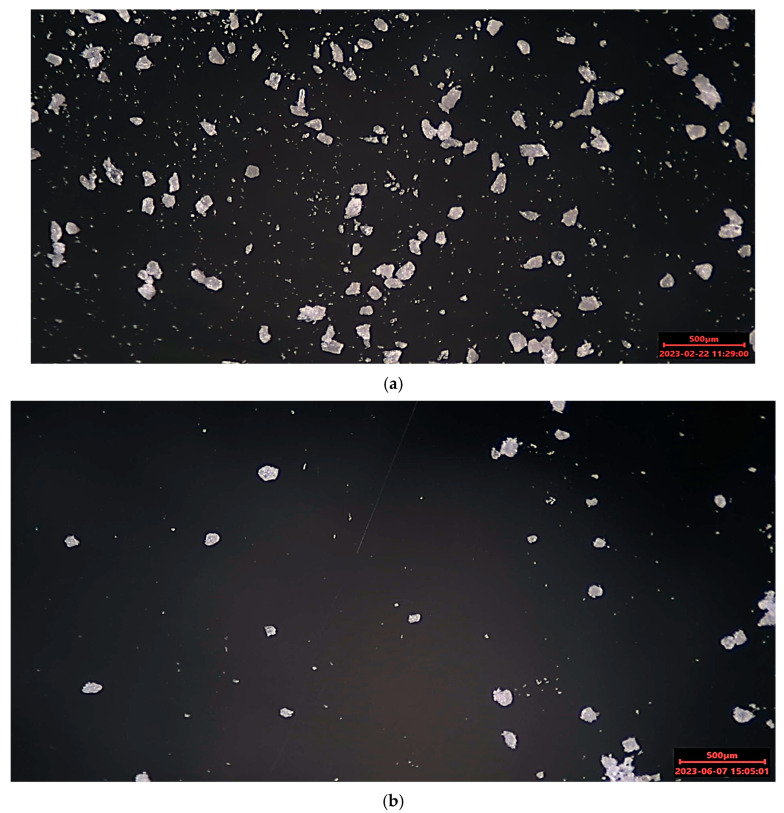

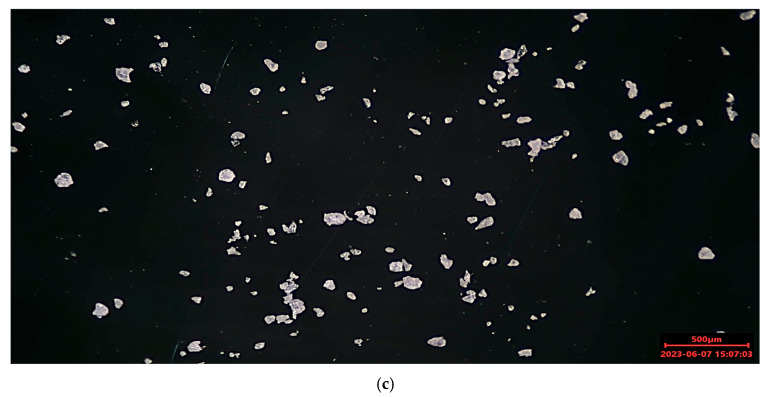
Optical microscope image of NOVUM: (**a**) Final powder of first grinding trial, Setting 1; (**b**) Final powder of second grinding trial; (**c**) Final powder of second grinding trial, Dedusted.

The powders produced in the technical center show a characteristic particle form. Due to the blunt impact of the material on the grinding disc, the particles show sharp edges (Figure 4). The particle form is similar for the powders of the first and second grinding trials.

## 5. Additive Manufacturing: High Speed Sintering

**Figure 5:**
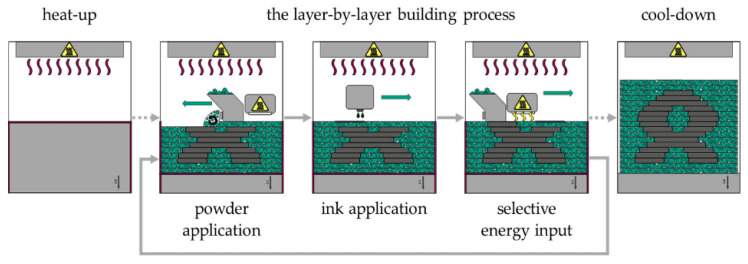
Schema of the High Speed Sintering process.

In the High Speed Sintering process, usually a powder of a semi-crystalline polymer is selectively melted by a sintering lamp using a Radiation Absorbing Material (RAM), which is selectively applied to the surface of the powder bed. The High Speed Sintering process is shown schematically in Figure 5. The heat-up is essential for processing the polymer powder. This is because the polymer powder must be brought to a temperature just below its phase transition temperature to avoid thermal disturbances in the process. In the first step of the layer-by-layer building process, the recoater applies a thin layer of polymer powder to the surface of the powder bed (powder application). A print head then selectively applies ink to the areas to be fused (ink application). The ink contains carbon black, which acts as a RAM. The sintering lamp is then moved across the powder bed surface (selective energy input). The temperature of the inked powder is raised above its melting point due to the RAM’s high absorption capacity. Due to the lower absorption of the radiation from the sintering lamp, the polymer powder not wetted by the ink does not fuse and remains loose, acting as a support. These steps—powder application, ink respective RAM application, and selective energy input—are repeated until all parts have been produced. Loose powder and parts must be cooled (cool-down) before parts can be removed from the powder bed [12].

### 5.1. Polymer Powder Analysis for High Speed Sintering

**Figure 6:**
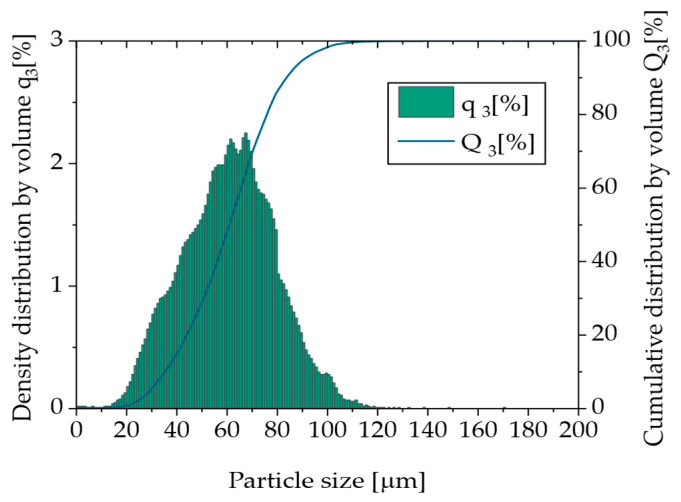
Volume-based particle size distribution of virgin Novum23 powder.

To obtain the particle size distribution (PSD) presented in Figure 6, dynamic image analysis according to ISO 13322-2 [13] was conducted using a Retsch Technology Camsizer XT. By using the X-Jet module with a dispersing pressure of 30.0 kPa and a gap width of 4.0 mm, virgin Novum23 powder was examined to classify the particle sizes from 0.0 µm to 200.0 µm in steps of 1 µm. The volume and number-based PSD curves were evaluated according to DIN ISO 9276 [14]. Dynamic scanning calorimetry (DSC) was conducted for virgin Novum23 powder between 25 °C and 240 °C with a heating and cooling rate of 10 K/min to derive the melting and crystallization behavior from the heating and cooling curves. Bulk density according to EN ISO 60 using a Coesfeld GmbH and Co KG (Dortmund, Germany) bulk density measurement device and tapped density according to DIN EN ISO 787-11 [15] using a J. Engelsmann AG JEL STAV II were measured using virgin Novum 23 to determine the Hausner ratio, which can be used to derive the flowability of a powder material. Microscopy of virgin Novum 23 was conducted using a KEYENCE Corporation VR-5000 to determine particle shape and surface properties of the polymer powder. An Emmeram Karg Industrietechnik MeltFlow@on (Karg Industrietechnik, Krailling, Germany) was used to determine the Melt Volume Rate (MVR) of virgin Novum 23 using a nominal load of 2.16 kg and temperatures of 200 °C, 220 °C, and 240 °C. UV-Vis spectroscopy was conducted for virgin Novum 23 powder using a Cary 5000 UV-Vis-NIR Spectrophotometer from Agilent Technologies Inc. (Santa Clara, CA, USA). The wavelength-dependent absorption spectrum of the powder was measured in the range between 250 nm and 2500 nm.

**Figure 7:**
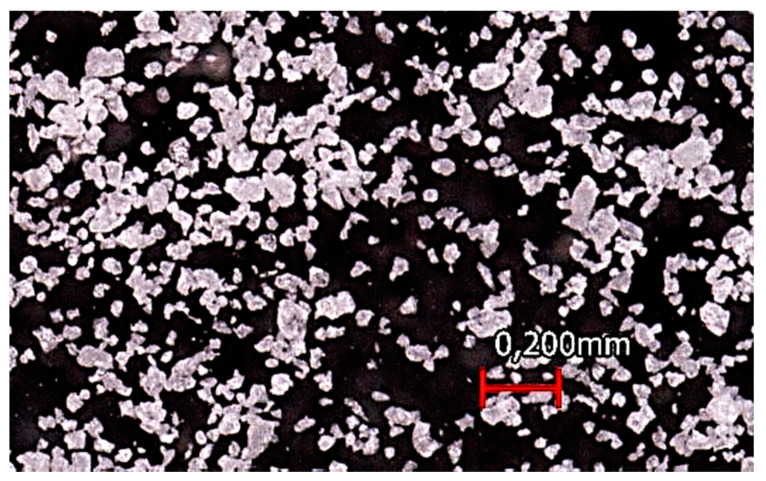
Microscopy image of virgin Novum powder.

**Figure 8:**
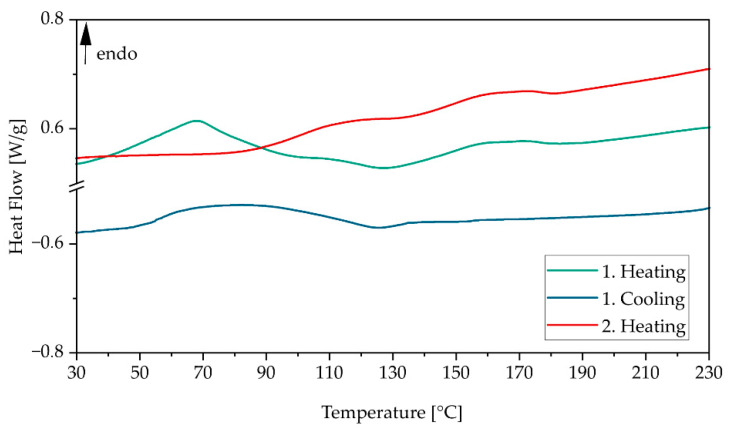
DSC heating and cooling curves of virgin Novum powder.

According to experience, a particle size distribution of approximately 20 to 80 µm and an aspect ratio (ratio of the smallest and largest diameter) of 1 of the particles is preferred for the High Speed Sintering. Figure 7 presents exemplary photo of the powder. Figure 8 shows the volume-based PSD curve of virgin Novum 23 powder. The Novum material shows a predominantly monomodal distribution with a more pronounced side towards small particle sizes. The characteristic values of the Novum 23 d10: 35.8 µm, d50: 61.4 µm, and d90: 84.2 µm are mainly within the specification and are presumably appropriate for the High Speed Sintering processes. Only 1.57% were bigger than the layer thickness of 100 µm.

**Figure 9:**
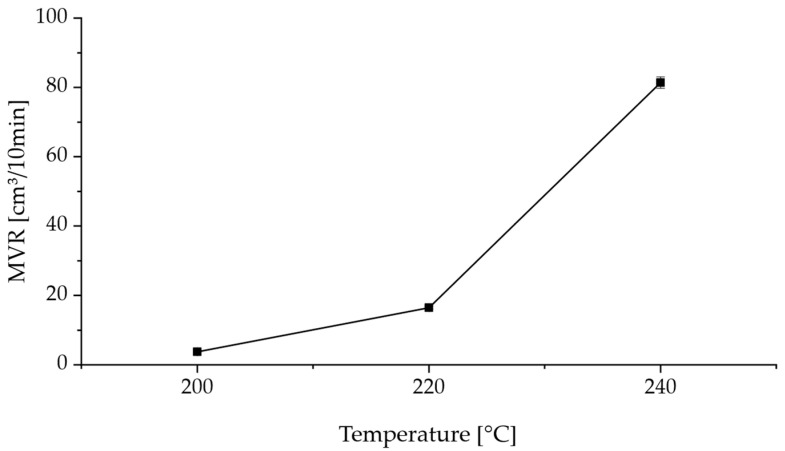
MVR values and their standard deviation at 200 °C, 220 °C, and 240 °C of virgin Novum23 powder.

The microscopy image of virgin Novum powder particles (Figure 9) shows that the powder particles are not perfectly spherical but tend to have a cryogenically milled character due to their partially sharp edges. According to Schmid [16], a low aspect ratio is desired to ensure powder flowability. The predominantly spherical character shown in the microscopy image suggests a low aspect ratio and thus a certain powder flowability. Based on the particle size distribution and the microscopy images, a theoretical processability within High Speed Sintering can be concluded.

Bulk and tapping density measurements of virgin Novum powder resulted in a bulk density of 0.46 g/cm^3^ and a tapping density of 0.62 g/cm^3^. By dividing the tapping density by the bulk density, a Hausner ratio of 1.35 for virgin Novum23 powder was calculated. The Hausner ratio is a measure of the flowability of powder material. According to Schmid [9], powders with a Hausner ratio between 1.25 and 1.4 are categorized as powders with reduced flowability. Despite the theoretically reduced flowability, the material was able to be processed within the High Speed Sintering process. Since the measuring method of the Hausner ratio is a static measurement, it is assumed that the recoating system, consisting of a vibrating blade (dynamic process), led to a certain fluidization of the polymer powder and increased the flowability due to vibration.

Figure 8 shows the heating and cooling curves of virgin Novum powder. Regarding the first heating (green curve), a bigger (~70 °C) and smaller phase transition (~150 °C) can be seen. Since the bigger melting peak disappears on second heating (red curve), it is assumed that the production of the polymer powder contributed strongly to the thermal prehistory of the material. Since the second heating shows no melting peak, which is typical for semi-crystalline polymers, it is assumed that Novum shows the behavior of an amorphous or semi-crystalline polymer. The glass transition temperature was calculated for both heating curves at 150 °C. In order to avoid sintering processes within the High Speed Sintering process, this temperature should not be exceeded based on the DSC measurement.

Regarding the first cooling (blue curve), a certain phase transition occurs at ~130 °C. As it is not clear from the curve whether it is a crystallization peak (semi-crystalline polymers) or a glass transition (amorphous polymers), the process temperature should not fall below this temperature in the High Speed Sintering process in order to avoid thermal effects such as curling or warpage. Based on the results of the DSC measurement, there is a theoretical processing window of 20 degrees for the process temperature between 130 °C and 150 °C. This is a much narrower range than in the case of well-known PA12 material, for which the processing window is 30–40 degrees.

Figure 9 shows the results of the MVR measurements of virgin Novum powder at 200 °C (3.7 cm^3^/10 min), 220 °C (16.5 cm^3^/10 min), and 240 °C (81.4 cm^3^/10 min). It can be seen that MVR increases with increasing melt temperature. Since the MVR value is a measure of the melt viscosity, it can be assumed that a higher MVR value equals a lower melt viscosity. As sintering is a time and temperature-dependent process, a lower melt viscosity can increase the sintering behavior of the polymer particles. Thus, regarding sintering, a higher part temperature should be aimed for using increased selective energy input.

**Figure 10:**
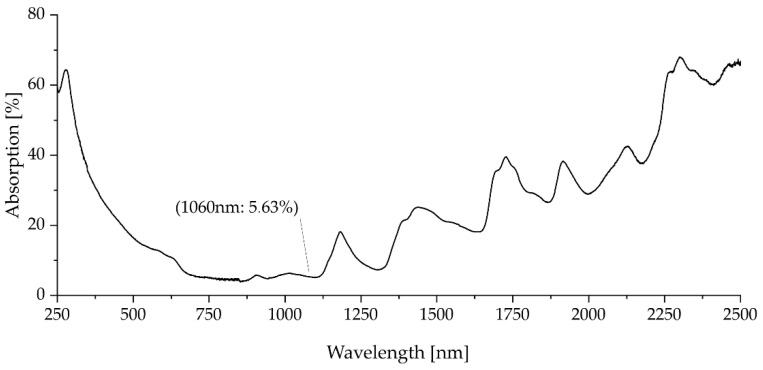
UV-Vis spectroscopy curve of virgin Novum powder.

Pezold [17] examined the emission spectrum of the sintering lamp of the VX200 HSS from voxeljet AG. It was shown that the sintering lamp has its maximum emission at maximum lamp power at 1060 nm. Figure 10 shows the wavelength-dependent absorption spectrum of virgin Novum23 powder as a result of the UV-Vis-spectroscopy. It can be seen that at a wavelength of 1060 nm, 5.63% of the incoming radiation from the sintering lamp would be absorbed by the polymer powder. In comparison, carbon black, as part of the ink used in High Speed Sintering, has an absorption greater than 90% over the entire wavelength range. The higher absorption of the radiation from the sintering lamp of the wetted polymer powder by the ink leads to the fusion of the powder particles. The unwetted areas of the polymer powder with lower absorption of the radiation from the sintering lamp remain loose.

### 5.2. Machine for High Speed Sintering

**Figure 11:**
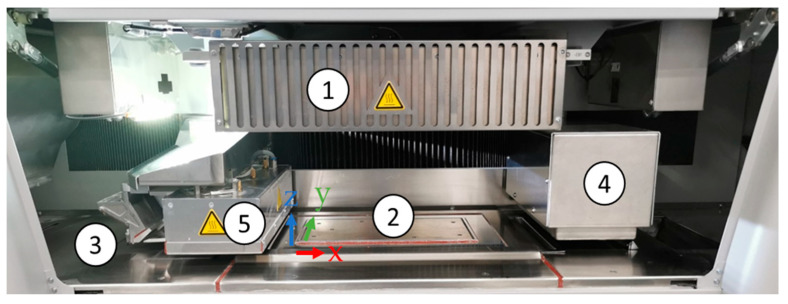
View of the build chamber of the voxeljet AG VX200 HSS: Overhead lamp (**1**), build box (**2**), recoater (**3**), printing module (**4**), and sintering lamp (**5**).

Figure 11 shows the build chamber of the Voxeljet AG VX200 HSS ‘beta version’, which was used to manufacture HSS parts. The overhead lamp (1 in Figure 11), containing six ceramic radiators, was used to reach the selected process temperature and to maintain a homogenous temperature distribution on the powder bed surface. The build box (2 in Figure 11) consisted of an in z-direction vertically movable and heatable floor plate, surrounded by four separately heatable side walls. The dimensions of the build box were 290 mm in x-, 140 mm in y-, and 180 mm in z-direction, which resulted in a maximum build volume of 7.308 cm^3^.

The layer thickness is defined by the stroke of the floor plate in the negative z-direction. A double-walled, fluid-heated, custom recoater (3 in Figure 11) flooded with heat-resistant silicone oil and heated by a Huber Ministat 230 was used to apply each layer of powder using a vibrating blade system. The powder output was determined by the recoater gap, the intensity of vibration, and the flow properties of the powder. Due to the design of the custom recoater, a homogeneous temperature of the powder output could be achieved. Further, the vibrating blade system of the recoater system enables the processing of particle sizes slightly bigger than the layer thickness.

The ink-printing module (4 in Figure 11), consisting of three XAAR 1003 print heads, which are arranged staggered in two rows, was connected to the machine’s fluid circulation system. For manufacturing, the Voxeljet AG ( Friedberg, Germany) HSS Ink Type B was used, containing carbon black as RAM. The sintering lamp (5 in Figure 11) was equipped with a 2 kW halogen lamp.

### 5.3. Manufacturing Process of High Speed Sintering

The parameters of Table 7 were used for manufacturing parts using High Speed Sintering. The process parameters used in this work are based on preliminary work with Novum, machine specifications, experience values, and the results of the polymer powder analysis for High Speed Sintering. While the Overhead lamp powder determines the process temperature of the powder bed, the sintering lamp power, greyscale, and sintering lamp speed determine the quantity and duration of the selective energy input. The greyscale value determines the amount of RAM that is applied per voxel (volume element) and thus the amount of energy that is induced into the polymer powder around.

**Figure 12:**
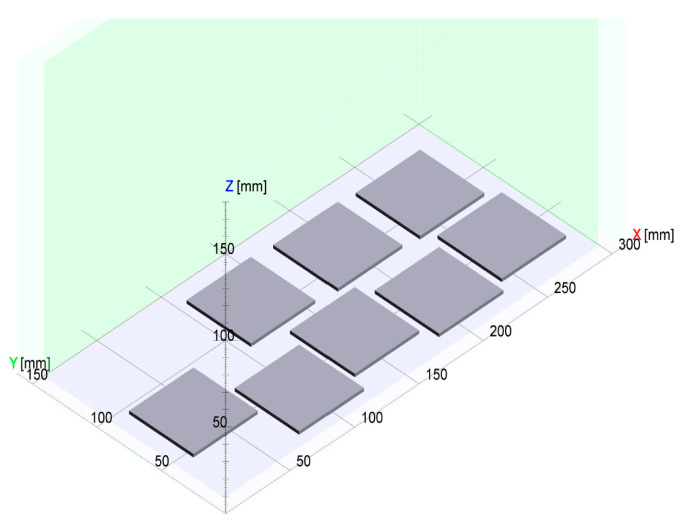
Layout of the High Speed Sintering build job: Eight insulation plates.

Figure 12 shows the layout of the High Speed Sintering build process, consisting of eight insulation plates to test the dielectric properties of Novum parts manufactured with High Speed Sintering.

After manufacturing, the parts were glass bead blasted within a post-process using a Normfinish DI12 injector blasting cabin from Leering Hengelo B.V., using a blasting pressure of 3 bar.

## 6. Selective Laser Sintering

**Figure 13:**
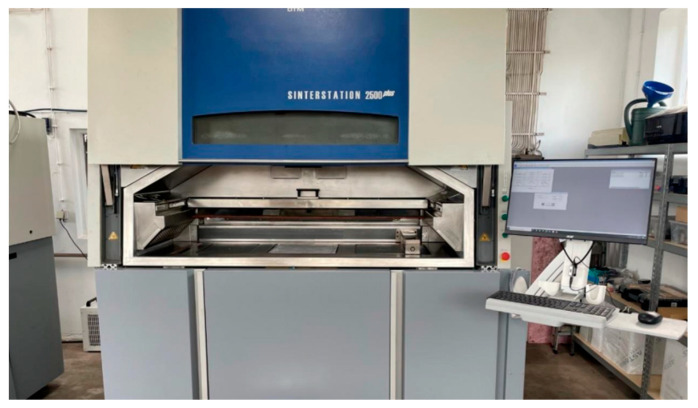
Sintering station.

The SLS-processability of NOVUM biomaterial has been tested on a Sinterstation DTM 2500 plus (Figure 13).

In order to obtain a proper sintering of the NOVUM powder, it was necessary to run a number of pre-heating, warm-up, build, and cool-down stages. During the printability test, it was observed that the rheology of the material appeared to be unsatisfactory. Although a high-energy recipe (40 W @ 0.1 mm scan spacing, single scan) has been used, the scanned surface did not melt sufficiently. During building, the adhesion of the material to the roller became a critical factor. While scanning, the appearance of smoke indicated material decomposition.

**Figure 14:**
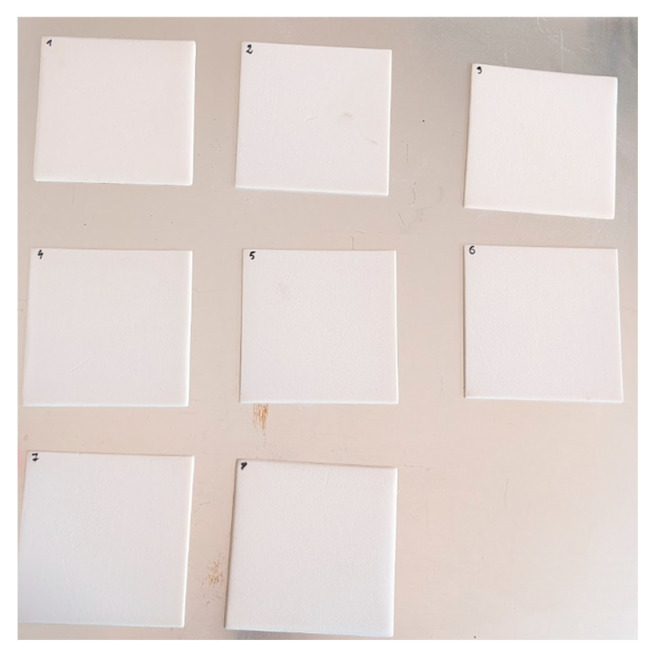
Samples printed on the sintering station.

For the printing of the final testing samples (Figure 14), the parameters are presented in Table 8, Table 9 and Table 10.

Summarizing, the material shows almost no noticeable curling during processing, whilst good processability shows an amorphous behavior. Since the required pre-heating temperature for this material is relatively low (over 10–12 K below its melting point), this prevents the fabrication of strong parts, as the rheology of the molten material remains insufficient even at very high laser-applied energy densities.

## 7. Electrical Properties Determination

As aforementioned, the main goal of these investigations was the comparison of the dielectric properties of the printed material using various additive manufacturing technologies. Electrical insulation systems operate under conditions of continuous exposure to electric fields, temperature fields, mechanical stress, and chemical reactions. Correct fabrication of individual insulation system components is crucial in terms of whole insulation operational reliability and long service life. In the case of high-voltage devices, the quality of components must be verified by electrical parameter measurements, especially when developing new production technologies. Production processes fundamentally affect the electrical parameters of given components compared to their basic forms. The production process can introduce dielectric or conductive impurities into the final component. Both types of defects have a different impact on the lifetime of the final product. The most undesirable defects are gas bubbles and sharp metallic particles. Both defects cause partial discharges that degrade the insulation system in the long term, ultimately leading to catastrophic failure. Other types of impurities increase the conductivity of the insulating material, causing increased material losses and a decrease in insulating capacity. The change in the conductivity of the insulation material is particularly significant in HVDC systems, where the distribution of the electric field in the insulation system depends on the conductivity of the materials used. Dielectric defects that affect the change of polarization phenomena in the insulation system also affect the increase in polarization losses and the change in electrical permittivity, which determines the distribution of the AC electric field.

Different printing technologies have different technological pros and cons, causing the introduction of defects into the basic form of the dielectric material, e.g., in the form of a matrix of a conductive nature, degradation of the dielectric material at elevated temperature, and introduction of air voids between successive applied layers. Basic electrical measurements such as determination of sample conductivity, determination of the electrical permittivity and polarization losses, determination of the partial discharges inception voltage and their form, and determination of the breakdown voltage of the sample allow for the assessment of the quality of the applied printing technology. These measurements can be carried out in accordance with the IEC and ASTM normative recommendations. Detailed information can be found in the relevant standards IEC 62631-3-1 [18], IEC 62631-3-2 [19] and IEC 61340-2-3 [20], IEC 60243-1 [21], IEC 60270 [22], D 149-09 [23]. Detailed descriptions of the measurement stands used in the investigations can be found in [5]. The KEYSIGHT B2987A electrometer and Resistivity Cell (16008B) were used to measure volume resistivity. The measurements of electric permittivity and dielectric loss factor were made using a Solartron 1260 Frequency Response Analyzer (West Sussex, UK) with a 1296 dielectric interface. The ICM System by Power Diagnostix (Aachen, Germany) was used to measure partial discharges. The dielectric withstand was measured in a flat electrode system fed by a TP60 test transformer equipped with HV measuring.

The measurements were performed on 10 × 10 cm flat samples which were conditioned and oil-impregnated. The conditioning process was performed to remove any moisture from the samples, the oil impregnation was performed to assess the possibility of using the components in oil insulation systems and to eliminate unwanted discharges during PD and breakdown voltage measurements.

The conditioning of samples was performed at reduced pressure of 10 mBar and ambient temperature of 100 °C for 8 h. Impregnation with synthetic oil was performed at a reduced pressure of 10 mBar and lasted for 6 h. The ambient conditions during the measurement were the following: temperature 21 °C, air humidity 24%, and pressure 999 hPa.

Three kinds of printing technologies were used for the fabrication of the evaluated samples, as follows:

- Extrusion printing technology
- High-speed sintering
- Laser sintering

The next paragraph presents the electrical parameters measurement results performed on flat samples developed with different technologies. During measurements, five samples were tested for every technology to determine the repeatability of developed samples; any important deviations were not observed. The measured parameters for the analyzed group of samples differed by 2.5%. Thus, for better clarification, the results present the measurements from a single sample. For laser sintering and for high-speed sintering, the results were compared to results measured on a sample before conditioning. The comparison of the results between raw form material and printed form sample for extruded printing can be found in [5].

### 7.1. Results for Samples from Extrusion Printing Technology

**Figure 15:**
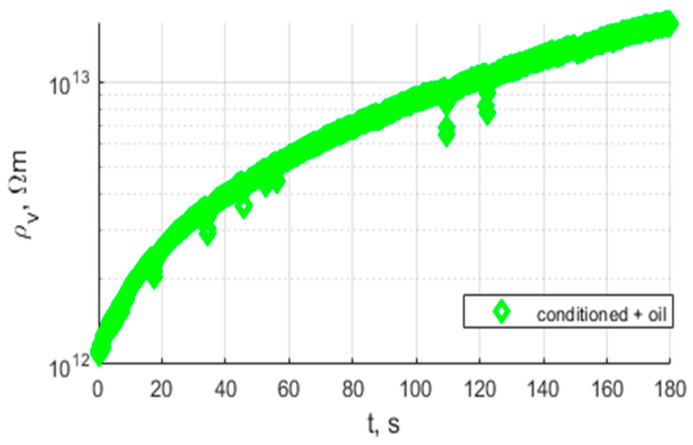
Characteristics of volume resistivity of the sample from extrusion printing.

Figure 15 presents characteristics of volume resistivity in time; this characteristic is obtained from the measurement of volume current at 1 kV DC and its recalculation based on the electrodes and sample geometry [11]. Volume resistivity characteristics contain information on the polarization process that appears in samples. For comparison purposes, every sample was measured in the same voltage conditions, and the acquisition time for volume resistivity was 180 s.

**Figure 16:**
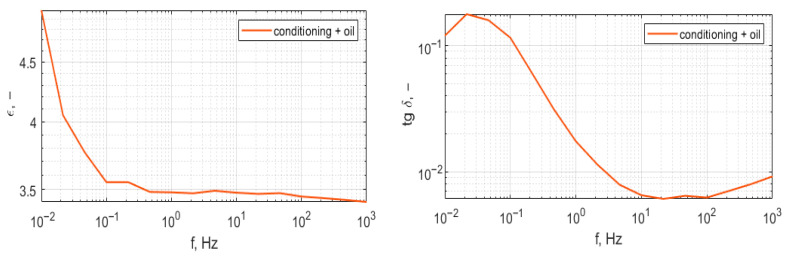
Characteristics of permittivity and dissipation factor of the sample from extrusion printing.

Figure 16 presents wideband permittivity and dissipation factor characteristics; the measurement frequency range was set from 1 mHz to 1 kHz.

### 7.2. Results for Samples from High-Speed Sintering

**Figure 17:**
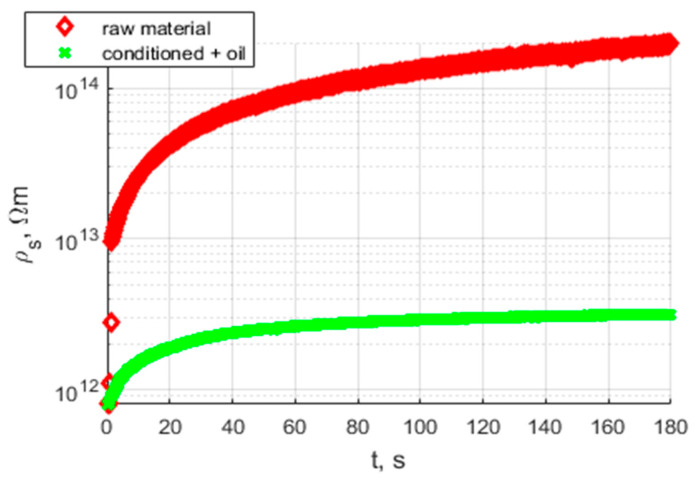
Characteristics of volume resistivity of the sample from High-speed sintering, raw sample, and after conditioning and oil impregnation.

Figure 17 presents a comparison between the raw sample before conditioning and the conditioned and oil-impregnated sample. The oil impregnation fills the air gaps in the porous structure of the sample and provides additional interfaces between the oil, dielectric material, and electrode.

**Figure 18:**
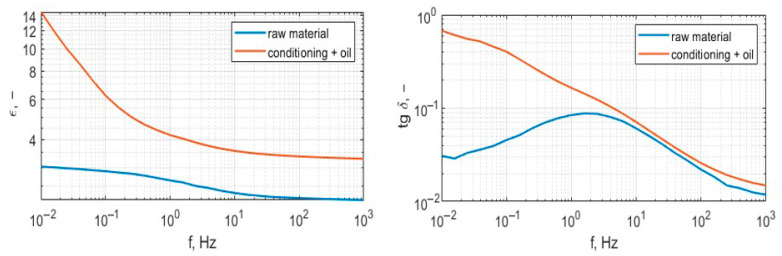
Characteristics of permittivity and dissipation factor of the sample from high-speed sintering, raw sample pre- and post-conditioning, and oil impregnation.

Figure 18 presents wideband permittivity and dissipation factor characteristics; the measurement frequency range was set from 1 mHz to 1 kHz.

### 7.3. Results for Samples from Selective Laser Sintering

**Figure 19:**
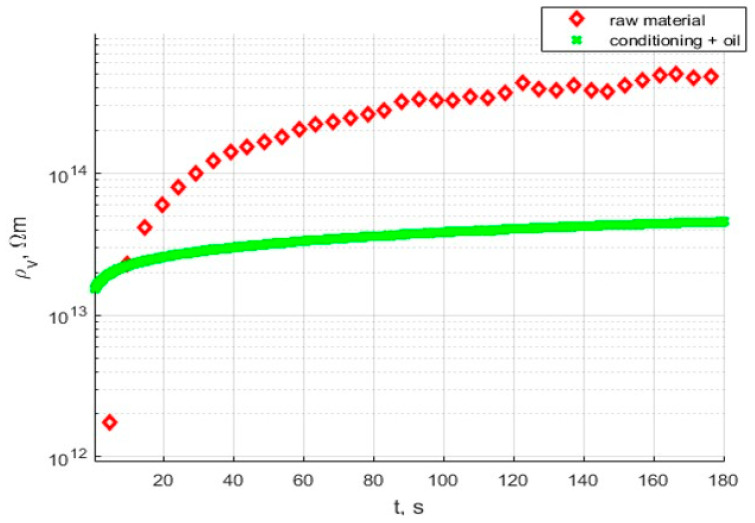
Characteristics of volume resistivity of the sample from Selective Laser Sintering, raw sample, and after conditioning and oil impregnation.

Figure 19 presents a comparison between the raw sample before and after conditioning and oil impregnation.

**Figure 20:**
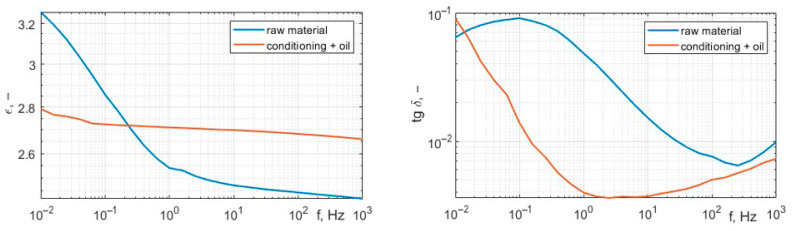
Characteristics of permittivity and dissipation factor of the sample from Laser Sintering, raw sample, and after conditioning and oil impregnation.

Figure 20 presents wideband permittivity and dissipation factor characteristics; the measurement frequency range was set from 1 mHz to 1 kHz.

### 7.4. Analysis of Obtained Results

Analysis of the results presented in Figure 15, Figure 17 and Figure 19 shows that the tested materials have volume resistivity greater than 10^12^ Ωm, which classifies them as electrical insulating materials. The conditioning process removes moisture from the sample, but only for these cases where material is porous (Figure 20), laser sintering, a drop of permittivity for lower frequencies is an indicator of moisture reduction [24,25]. The samples produced with the extrusion printing and High Speed Sintering methods are characterized by the rise of the permittivity for low frequencies, which can be an indicator of higher moisture trapped inside the material. The oil fills the voids in the material and provides interfaces for Maxwell-Wagner polarization effects [26,27]. Samples made with High Speed Sintering technology were based on a matrix containing the carbon filler, which caused the drop in the volume resistivity by two orders of magnitude after conditioning and oil impregnation. The oil impregnation in this case multiplies the paths of the current from HV electrodes bridging conducive parts of the sample; this effect is revealed for volume resistivity measurements and the measurement characteristics of permittivity and dissipation factor. The increase in values for lower frequencies is visible in Figure 19.

The comparative results were collected in Table 11, Table 12 and Table 13.

The last part of the evaluation of the dielectric properties of the printed sample was to determine the partial discharge inception voltage and breakdown voltage. The procedure for determining PDIV covered a constant increase of the applied voltage up to 40 kV. When partial discharges started to appear, voltage was noted as PDIV; when no PDs were generated before breakdown, the sample was treated as PD-free. The comparison of results is presented in Table 13; the breakdown voltage covers the mean value of the obtained results and its standard deviation, and the PDIV represents the minimal value in the analyzed group of samples.

**Figure 21:**
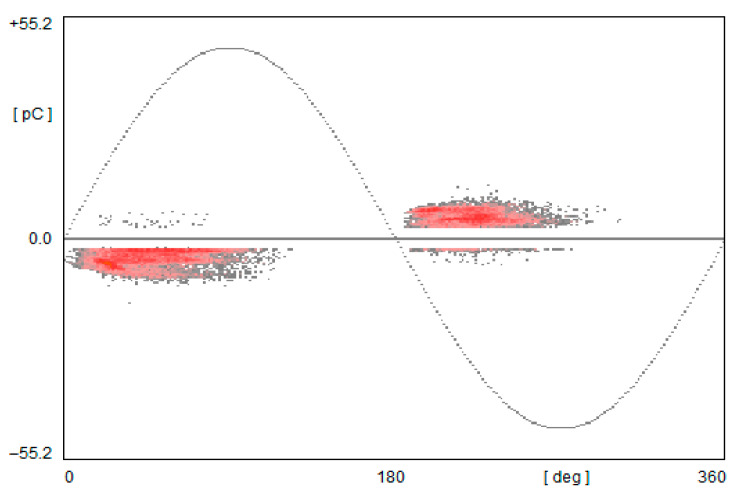
Exemplary recorded PRPD patterns in a sample from High Speed Sintering during PD measurements, a conditioned and oil-impregnated sample.

The samples made by laser sintering have no sources of partial discharges, meaning that the oil impregnation covered the whole air voids that could appear in the material during the fabrication process. The extrusion printing and high-speed sintering samples were characterized by the appearance of Partial Discharges. The laser-sintered sample showed a low level of PDIV; the extruded sample presented PDIV near the breakdown voltage [5]. Appearance of partial discharges below the nominal voltage caused constant degradation of the insulation system. Figure 21 presents the phase-resolved partial discharges (PRPD) patterns measured for the high-speed sintered sample at the Partial Discharge Inception voltage.

The PRPD patterns indicate the gaseous voids inside the tested samples; the same issue was reported for the extruded sample [5].

The breakdown field was the highest for the samples made with the extrusion printing, with a breakdown electrical field more than twice as much as that field obtained for laser-sintered samples.

## 8. Conclusions

The results obtained demonstrate new directions in the commercialization of additive manufacturing with a new NOVUM biomaterial in high voltage insulation. It has been confirmed that such material can be printed using all the investigated technologies (FDM, HSS, and SLS), although due to its amorphous nature, the sintering process is deemed challenging. In case of extrusion-based additive manufacturing, most of the dielectric properties are promising, but the microstructure of the material can lead to the generation of partial discharges. For High Speed Sintering technology, the dissipation factor and volume resistivity disqualify the material for application for high voltage insulation. This is probably the effect of conductive particles (carbon black) in the ink used. Selective Laser Sintering gives PD free structures; however, due to low mechanical performance, industrial application is still questionable. Porosity in insulation materials has a significant impact on partial discharge (PD) activity. Air-filled voids and cavities within the insulation act as localized regions of electrical stress concentration, which can initiate and propagate PD phenomena. The introduction of a suitable impregnating agent into these voids can enhance both the electrical and mechanical performance of the insulation. In the tests conducted, the printed material was impregnated with transformer oil. Samples produced via the SLS process were readily impregnated, indicating good permeability. In contrast, extrusion-based printed samples exhibited trapped voids, which hindered effective oil penetration.

Table 14 presents a comparison of all tested additive manufacturing technologies considering various parameters/functionalities.

Future work will focus on enhancing the print quality and post-processing treatments for FDM-printed parts to meet dielectric reliability standards. As mentioned earlier, in that process, the voids in the material are built, reducing the adhesion between the layers, which results in weaker dielectric properties (creation of partial discharges) and influencing mechanical stability. To overcome those problems, the application of isostatic pressure is considered. Also, the usage of more controllable extruders will be investigated.

## Figures and Tables

**Table 1:** Analytical Data of the first grinding trial at Dressler with NOVUM.

Property	Test Method	Value	Unit	Target	NOVUM		
					Setting 1	Setting 2 (Coarse)	Setting 3 (Fine)
Particle size distribution	Laser diffraction	D10	µm	-	12	13	11
		D50	µm	50–60	57	64	49
		D90	µm	-	110	121	105
	Air classifier sieve	<32 µm	w%	-	19	19	25
		<63 µm	w%	-	62	54	69
		<100 µm	w%	-	100	100	100
		<160 µm	w%	-	100	100	100
Moisture	Kern DBS 60-3	-	w%	-	1.31	1.37	1.43

**Table 2:** Analytical Data of the second grinding trial at Dressler with NOVUM.

Property	Test Method	Value	Unit	Target	NOVUM		
					First Grinding Trial Setting 1	Second Grinding Trial	Second Grinding Trial Dedusted
Particle size distribution	Laser diffraction	D10	µm	20–40	12	10	37
		D50	µm	30–60	57	52	69
		D90	µm	90–110	110	107	111
		Q10 µm	%	-	6	10	0.5
	Air classifier sieve	<32 µm	w%	-	19	28	12
		<63 µm	w%	-	62	62	50
		<100 µm	w%	-	100	100	100
		<160 µm	w%	-	100	100	100
Moisture	Kern DBS 60-3	-	w%		1.31	0.95	0.93

**Table 3:** Powder properties of the first grinding trial at Dressler with NOVUM-23.

Property	Test Method	Value	Unit	NOVUM-23		
				Setting 1	Setting 2 (Coarse)	Setting 3 (Fine)
Apparent density	DIN EN ISO 60	-	g/L	379	370	365
Hausner ratio	ASTM D1895	500 taps	-	1.35	1.34	1.38
Funnel test	DIN EN ISO 6186	A_15 mm_	s	-	-	-

**Table 4:** Powder properties of the second grinding trial at Dressler with NOVUM.

Property	Test Method	Value	Unit	NOVUM		
				First Grinding Trial Setting 1	Second Grinding Trial	Second Grinding Trial * Dedusted
Apparent density	DIN EN ISO 60	-	g/L	379	440	451
Hausner ratio	ASTM D1895	500 taps	-	1.35	1.34	1.27
Funnel test	DIN EN ISO 6186	A_15 mm_	s	-	-	15 *

* By tapping the funnel once.

**Table 5:** Spreadability measurement conducted via MDK of the first grinding trial at Dressler with NOVUM.

Layer Thickness	500 µm	
Condition	25 °C	80 °C
Setting 1	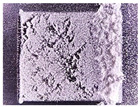	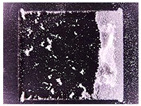
	Poor spread, 88% coverage	Not spreading, 31% coverage
Setting 2 (coarse)	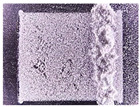	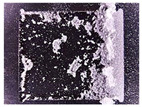
	Poor spread, 95% coverage	Not spreading, 41% coverage
Setting 3 (fine)	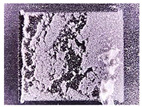	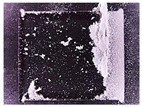
	Poor spread, 76% coverage	Not spreading, 33% coverage

**Table 6:** Spreadability measurement conducted via MDK of the second grinding trial at Dressler with NOVUM.

Layer Thickness	100 µm
Condition	80 °C
Second grinding trial	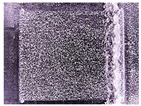
	Fair spread, 66% coverage
Second grinding trial Dedusted	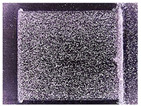
	Average spread, 56% coverage

**Table 7:** Used process parameters of the VX200 HSS.

Parameters	Values	Units
Powder ratio (used/virgin)	0/100	%
Temperature hysteresis	5	°C
Build box wall temperature	80	°C
Build box floor plate temperature	130	°C
Recoater temperature	100	°C
Recoater speed	0.09	m/s
Recoater gap	3.0	mm
Recoater vibration strength	90	%
Layer thickness	100	µm
Sintering lamp power (SLP)	100	%
Sintering lamp speed (SLS)	0.11	m/s
Overhead lamp power	23	%
Process temperature (PT)	145	°C
Ink print head speed	0,39	m/s
Greyscale (GS)	36 (GS6)	pL/voxel
Pre-layers	30	-
Post-layers	30	-

**Table 8:** Warm-up stage parameters.

Parameter	Unit	Stage Height z-Height Start	Setpoint
Maximum oxygen content	%	0	5.5
Part heater setpoint	°C	0	100
Left/Right feed heater setpoint	°C	0	60
Part cylinder heater setpoint	°C	0	100
Piston heater setpoint	°C	0	100
Powder layer thickness	mm	0	0.12

**Table 9:** Build stage parameters.

Parameter	Unit	Stage Height z-Height Start	Setpoint
Maximum oxygen content	%	0	5.5
Part heater setpoint	°C	0	100
Left/Right feed heater setpoint	°C	0	60
Part cylinder heater setpoint	°C	0	100
Piston heater setpoint	°C	0	100
Powder layer thickness	mm	0	0.12
	**Part parameters**		
Fill scan speed	mm/s		10.16
Fill laser power	W		45
Slicer fill scan spacing / hatch distance	mm		0.13
Fill scan count	number		2 (double scan)
Outline scan speed	mm/s		1.778
Outline laser power	W		12
Laser beam diameter @ part bed	μm		420

**Table 10:** Cool-down stage parameters.

Parameter	Unit	Stage Height z-Height Start	Setpoint
Maximum oxygen content	%	0	5.5
Part heater setpoint	°C	0	110
Left/Right feed heater setpoint	°C	0	60
Part cylinder heater setpoint	°C	0	110
Piston heater setpoint	°C	0	110

**Table 11:** Comparison of volume resistivity Ωm determined for the tested samples.

Material	Extrusion	High Speed Sintering	Laser Sintering
Raw	-	3.14 × 10^14^	5.41 × 10^14^
Conditioned and oil impregnated	2.22 × 10^13^	2.69 × 10^12^	5.11 × 10^13^

**Table 12:** Comparison of relative permittivity and dissipation factor determined for the tested samples at 50 Hz.

Material	Extrusion	High Speed Sintering	Laser Sintering
Raw	-	2.23 → 0.027	2.44 → 0.008
Conditioned and oil impregnated	3.48 → 0.0064	3.4 → 0.031	2.69 → 0.004

**Table 13:** Comparison of minimal Partial Discharge Inception Voltage and mean Breakdown Field determined for the tested samples.

Material	Extrusion	High Speed Sintering	Laser Sintering
PDIV, kV	33.6	4.13	PD free
Breakdown Field, kV/mm	13.57 (σ = 1.04)	16.96 (σ = 2.2)	12.54 (σ = 1.34)

**Table 14:** Comparison of all tested additive manufacturing technologies for the processing of biomaterials.

Parameter	Extrusion	High Speed Sintering	Laser Sintering
Print quality	Moderate	Low	Moderate
Processing challenges	Low	Low	Moderate
Mechanical stability	High	Low	Low
Scalability (printing of large parts)	High	Moderate	Moderate
Suitability for high voltage applications	High	Low	Moderate

## Data Availability

Data are contained within the article.
